# My Memory of Walter Fitch (1929–2011) and Starting *Molecular Biology and Evolution*

**DOI:** 10.1093/molbev/msu133

**Published:** 2014-05-02

**Authors:** Masatoshi Nei

**Affiliations:** ^1^Institute of Molecular Evolutionary Genetics and Department of Biology, Pennsylvania State University

**Keywords:** Walter Fitch, molecular biology and evolution

More than 3 years have passed after Walter Fitch died on March 10, 2011. However, my memory of Walter is still fresh and vivid. From the time when he and I started this journal *Molecular Biology and Evolution* (MBE) in 1983, we had a close contact for about 15 years. He and I also started the *Society for Molecular Biology and Evolution* (SMBE) in 1992. We had a common interest in the theoretical study of molecular evolution and phylogenetics, and we often met in scientific meetings. In this article, I would like to discuss his contributions to the development of MBE and SMBE as well as a few of his research accomplishments concerning molecular evolution. A more general story of his life and science is written by Atchley (2011), Ayala (2011), Hall (2011), and McClure (2011).

I met Walter for the first time in a symposium on population genetics and evolution organized by Newton Morton in 1972 at the University of Hawaii in Honolulu. He was still young with dark hair and very energetic. He presented his collaborative study with Chuck Langley concerning a statistical analysis of molecular clocks with protein data (Langley and Fitch 1973). They used the amino acid sequences of hemoglobins, cytochrome c, and fibrino-peptides, the only protein sequences available for many organisms at that time. This study showed that the number of amino acid substitutions does not increase linearly with time. However, Walter later concluded that the deviation from the linearity is not great and we should not abandon the idea of molecular clocks (Fitch 1976). He was a pragmatic man.

He published several papers on molecular clocks, but this was not the subject of his main interest. His main interest was in the construction of phylogenetic trees from molecular data though he published many papers on other subjects of molecular evolution. In fact, the most famous paper written by him is the one published by Fitch and Margoliash in *Science* (1967). In this paper, the authors presented the first phylogenetic tree of a sizable number of animal and fungal species by using a single protein, cytochrome c. This was a revolutionary paper and later became a Citation Classic in *Current Contents* (1988). In this study, they developed a new statistical method of tree-making using a least-squares criterion. This paper together with Cavalli-Sforza and Edwards’ (1967) ushered the new era of molecular phylogenetics.

His contribution to phylogenetics is not confined to this paper. Later, he developed a basic algorithm for the parsimony method of tree construction using nucleotide sequence data (Fitch 1971). This algorithm is now widely used as a tool of parsimony tree construction, and this paper also became a Citation Classic in 1987. In 1981, he proposed a nonsequential method of phylogenetic construction using distance data. This work later influenced the development of neighbor-joining method of Saitou and Nei (1987). During this period, there was a highly contentious debate between so-called cladists and pheneticists in the area of systematic biology, but Walter did not participate in this philosophical debate. His interest was in the development of technically efficient methods of phylogenetic tree construction. He once told me that around this time he was an associate editor of the journal *Systematic Zoology* (now called *Systematic Biology*) but he was fired from the position in 1980 because he was not a real cladist.


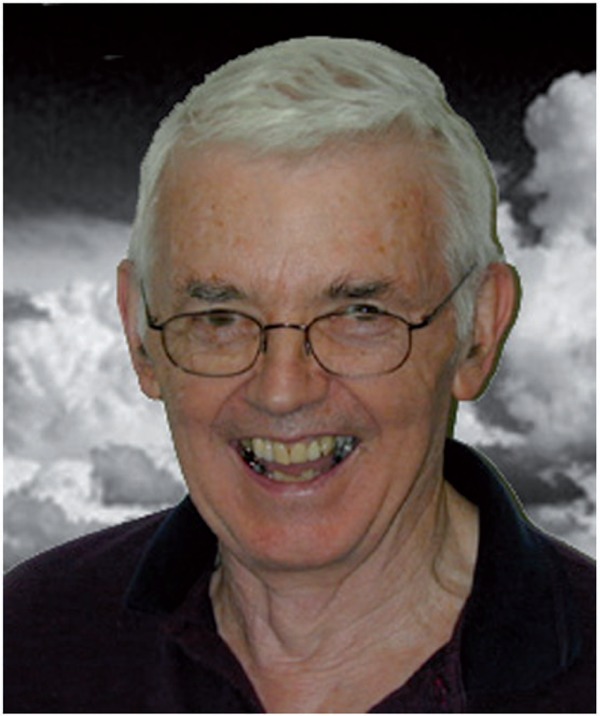


Another contribution of Walter’s to evolutionary biology was his proposal of new concepts or technical terms that are useful for data analysis. One of them was the distinction between orthologous and paralogous genes when duplicate genes exist (Fitch 1970). This distinction has become essential in the study of molecular evolution, and many investigators are now using the word “orthology” or “orthologous” without knowing their origin. He also defined the word homology as an all-or-none concept. He insisted that homology should not be used as a measure of the extent of sequence similarity of two homologous genes. He argued that “homology” should be used only for distinguishing between homologous and analogous characters as was done in classical biology.

Molecular evolutionary biology was born in the 1950s and 1960s when a small number of molecular biologists started to study the evolutionary changes of amino acid and nucleotide sequences between different species. In the mid-1960s, population geneticists entered into this field by initiating the study of electrophoretic variations of proteins within and between populations. These two lines of study of molecular evolution were conducted almost independently by molecular biologists and population geneticists, respectively, until around 1970. At this stage, a new journal called *Journal of Molecular Evolution* was launched by Springer Verlag with the editorship of Emile Zuckerkandl. Walter and I then contributed several papers to this journal. However, we realized that there were some problems with this journal. First, this journal was so expensive that many universities did not subscribe to it. Consequently, the papers published in this journal were not read widely. Second, because the journal was for a relatively small number of investigators, the quality of the papers was not necessarily high, and it took a long time for reviewing process. For these reasons many investigators were not very happy with this journal, and I started to consider the creation of a new journal that would be useful for both molecular biologists and population geneticists.

At this time, I received a letter from a commission editor of the publisher Alan Liss (later merged with John Wiley Publ. Co.), who asked me whether I could edit monographs on various subjects of molecular evolution periodically. It was in early 1982. However, I did not think that publication of such monographs would contribute greatly to the progress of molecular evolutionary biology. Instead, I thought that the creation of a new journal for publishing original papers in an inexpensive way would be more important. Fortunately, the commission editor enthusiastically supported my counter proposal. However, to start a new journal, I had to have agreements from my fellow evolutionists.

I therefore called or wrote to a dozen or two of key molecular evolutionists. I then received enthusiastic support from most of them, particularly from Walter Fitch. The next step was to make a list of potential editors and editorial board members. Because I was familiar with both molecular evolution and population genetics, this was not a difficult job. However, I was still unsure whether we could get a sufficient number of papers to launch a new journal. For this purpose, I needed support from young investigators as well as from established scientists. Fortunately, I had a plan to organize an international symposium on “Evolution of Genes and Proteins” (Nei and Koehn 1983) at the State University of New York, Stony Brook, in conjunction with the joint meeting of the Society of Study of Evolution and the American Society of Naturalists, June 1982. I therefore decided to have a special meeting concerning the inauguration of a new journal during this meeting. In this meeting, a sizable number of evolutionists gathered and approved the inauguration of a journal. It was also decided that Walter Fitch and I should be responsible for starting the journal. This discussion was continued in the dinner party sponsored by the Alan Liss. At the end of these discussions, we agreed that the new journal should be a vehicle to 1) have better communication between molecular biologists and population geneticists and 2) rapidly publish high-quality papers and that 3) the journal be owned and controlled by a scientific community and 4) it should be available to an international readership at an affordable price.

After the Stony Brook meeting, Walter and I were very busy deciding the name and purpose of the journal and finding an appropriate publisher of the journal. I felt some obligation for choosing Alan Liss, but Walter said we should not be bought by just one dinner. We then solicited the publication proposal from several publishers and finally chose the University of Chicago Press (UCP). As mentioned earlier, we had already decided that the new journal should be owned by investigators rather than the publishing company. This meant that we could change the publisher when their service is not satisfactory for us. In practice, we did not have any formal society of molecular evolutionists at that time, but we could form a small society consisting of the editors and editorial board members. UCP’s proposal was most appropriate for our purpose. This publisher was also doing well in publishing *American Naturalist* at that time. Some publishers offered an honorarium to the editors, but we rejected these publishers because Walter and I thought we were doing public service and should not have personal gain by serving as editors. On March 5, 1983, we finally signed the contract of the publication of MBE with UCP.

However, the decision about the publisher was only one of the tasks for starting a new journal. We had to decide the roles of editors and editorial board members and how to attract good papers. We had already decided that Walter and I should jointly control the journal, but I thought I should concentrate more on policy-making of the journal including the appointment of editorial board members and evaluating the performance of the publisher, whereas Walter concentrated on the quality control of published papers. I knew that Walter was energetic and could take care of detailed aspects of manuscript editing and publication. For this reason, Walter became the editor-in-chief and I took the job of managing editor. In practice, however, we did essentially the same work in editing and accepting manuscripts. We also appointed several associate editors who could handle reviewing and accepting of submitted papers. In addition, we appointed a few dozens of editorial board members whose primary job was to review manuscripts assigned by the editors or the associate editors.

Our next problem was how to collect high-quality manuscripts. For the first issue of the journal, we were supposed to have manuscripts from the researchers who attended the Stony Brook meeting, but their submission was slow although we promised quick publication. We also wanted to have the first paper of the first issue from a prominent molecular biologist, but his submission was delayed too much to be included in the first issue. Fortunately, Walter unexpectedly received an interesting paper on protein evolution by Max Perutz, a Nobel Laureate. We now decided to publish this as the lead paper of the first issue. This paper undoubtedly enhanced the visibility of the new journal MBE.

However, we had to make a somewhat unusual decision about publishing the first volume of MBE. In the Stony Brook meeting, we promised that the papers submitted to MBE would be published rapidly and volume 1 would be completed in 1983. However, at the beginning of 1983 it was obvious that we could not do it because we had not received enough manuscripts. Yet, we did not want to disappoint the authors who had submitted their papers in 1982. So, we decided to publish the first issue of volume 1 in December 1983, but the remaining five issues in 1984. The last issue of volume 1 was published in November 1984. In 1985, a favorable review of volume 1 of MBE appeared in *Nature* (Leigh Brown 1985). (At that time, a new journal was sometimes reviewed in *Nature*.) MBE also received an award (Honorable Mention) for excellence in publishing for 1984 from the Professional and Scholarly Publishing Division, Association of American Publisher. We were pleased with these favorable evaluations.

At this point, I should mention that both Walter and I worked hard to start a new journal but Walter continued his hard work even after the journal was firmly established. As I mentioned elsewhere (Nei 1994), he devoted nearly 50% of his time to MBE during the 10-year period of his editorship. He once told me that he read every paper published in MBE when he was the formal editor. Actually, he often found errors in the manuscripts accepted by other editors and then asked the authors for further revision. In reviewing manuscripts, he was careful enough to read all figure and table legends to which many other editors did not pay much attention. I have never heard any other editor who was so dedicated to editorial work.

According to Barry Hall (1994), I was also dedicated to editing MBE papers. He stated that in the first 10 years of journal production Water handled 186 papers and I handled 116 papers, the remaining 153 papers being handled by 15 associate editors. (This occurred partly because at that time the editors and associate editors were supposed to handle only the papers he or she received.) This was true, but Walter took care of proofreading of all papers and production of the journal. Therefore, Walter’s hard work and leadership was the important factor for producing a high-quality journal. According to the ISI citation statistics, the impact factor of MBE rapidly increased during the first 10 years and had the highest score in the category of evolutionary biology in 1991. The total number of pages published per volume also increased from 507 in 1984 to 1,410 in 1993. Of course, this growth was partly due to the fact that the research area of molecular evolution expanded enormously in this period and many investigators contributed their important papers to MBE.

Around 1990 Walter and I discussed the possibility of establishing a full-fledged society of MBE and our retirement from the editorial office. Later, we agreed that this should be done when we would complete the first ten volumes of MBE. For this purpose, I thought that we should have a symposium on molecular evolution and use this opportunity to establish a formal SMBE. This symposium was held at the Pennsylvania State University in June 1992. Fortunately, I received a grant of $30,000 to organize this symposium from the Alfred P. Sloan Foundation so that we could invite many world authorities on molecular evolution, including Lee Hood, Walter Gilbert, Roy Britten, and Giorgio Bernardi. More than 300 people gathered at the 3-day symposium.

The symposium was a memorable one, partly because there was a heated debate between Walter Gilbert supporting the early-intron hypothesis and Jeffrey Palmer proposing the late-intron hypothesis (Gibbons 1992). However, Walter Fitch and I were more concerned with the inauguration of SMBE. When the symposium started, we had not seriously considered the potential officers of the Society and the next editor. Therefore, Walter and I had to think about this problem as well as the scientific conference. At the end, however, we came up with the proposal of President Walter Fitch, President-elect Masatoshi Nei, Secretary and Treasurer Linda Maxson, New Editor Barry Hall, and Interim Councilor Caro-Beth Stewart. This slate was approved almost unanimously among the attendants at the inauguration meeting, and it was decided that the society should serve for the international community of molecular evolutionists.

However, this was the start of real organization of the society. We had to establish by-laws of the society, membership fees, first-year budget, and annual meetings, invitation of speakers for the first SMBE meeting, etc. SMBE is probably one of the first scientific societies in which email communication was used effectively to speed up the decision of the council meeting. I remember that the by-laws were revised several times and this was all done by email communication. As the first president of the society, Walter took a leadership for organizing a new society and producing various operating procedures. At this time, the Sloan Foundation gave us another grant of $60,000 as start-up funds for SMBE. This was a blessing because the income for SMBE came only from the sale of the journal and the membership fees, which were kept low as much as possible. At that time, there was no page charge for the authors and no color plate charge.

Walter suggested that the first SMBE meeting should be held at the University of California at Irvine (UCI), and all council members agreed with him. He then proposed that we should have a special session for graduate students and give an award of $500 to the student who gave the best presentation and that he would donate the money. The council members said that his donation was not necessary because we had a sufficient amount of money. However he insisted his contribution and pledged that he would continue his donation until his retirement from his university, UCI. To appreciate his generosity, the council decided to call the award the Walter Fitch Prize, and set up an endowment of $10,000 for the prize money in 1995.

The first SMBE meeting at UCI did not attract many attendants apparently because the society was not well known at that time. Yet the papers presented were of the first rate and there were foreign delegates and attendants, as expected for an international society. Walter was in charge of almost every aspect of the meeting. The second SMBE meeting was held in Athens, Georgia, in 1994, in conjunction with the meetings of the Society for the Study of Evolution, the Society of American Naturalist, and the Society for Systematic Biology. Partly because of the joint meetings, a large number of people attended this meeting. The first SMBE meeting outside the United States was held in Hayama, Japan, under the auspices of Naoyuki Takahata. This was really the first international meeting for SMBE and was attended by many people from various countries. Here, I should mention that this meeting was partially supported by a binational meeting support grant ($59,810) from the US National Science Foundation (NSF) for promoting the collaboration between young investigators from the United States and Japan (Nei and Takahata 1996). Actually, this grant was given to us from NSF with their initiation after they heard about our upcoming meeting in Japan. From then on, the annual SMBE meeting was held both in the United States and outside the United States. In these days the number of attendants was usually a few hundred, and I never expected that the annual meeting would later attract more than 1,000 people as it does now.

The MBE journal has also grown enormously. It now publishes about 4,000 large-size pages per year, and the rejection rate is as high as 73% (Kumar 2013). (In the first ten volumes, it was about 50%.) The impact factor has also risen to 10.4 in 2013. The publisher of MBE has changed from UCP to Allen Press in 1996 and then to Oxford University Press in 2003. During this period, Simon Easteal, Bill Martin, Marcy Uyenoyama, and Sudhir Kumar (current) have served as editor of MBE. Furthermore, a new journal *Genome Biology and Evolution* sponsored by SMBE was launched in 2009 with the editorship of Bill Martin. This journal publishes genomics-related papers, expanding the territory of SMBE enormously.

After retirement from the SMBE activity, Walter Fitch concentrated on his research project on evolutionary changes of influenza virus genes. In 2000, he published a statistical method for predicting the evolution of human influenza A (Fitch et al. 2000). In his study of evolution of influenza viruses, he used phylogenetic analysis by using his own computer programs based on parsimony methods, with which he was most comfortable. In his later years he had various ailments and retired from the university in 2009, but he continued to work even after the retirement. I should also mention that Walter had a long-term interest in the arguments against creationism, and after his retirement he wrote a book on this subject with the title of *The Three Failures of Creationism: Logic, Rhetoric, and Science*. This book was posthumously published from the University of California Press in April 2012. Unfortunately, I have not had a chance to read it, but I am glad that he could finish the book before he died. I heard that he passed away peacefully in his sleep in the early morning of March 10, 2011. He was a man of creativity, generosity, hard-working, and conscientiousness.

For his outstanding contributions to evolutionary biology, he received many awards and recognitions including the membership for the National Academy of Sciences, American Academy of Arts and Sciences, American Philosophical Society, and foreign member of Linnean Society of London, and honorary doctorate of North Carolina State University.
